# Diffuse Leptomeningeal Glioneuronal Tumour with 9-Year Follow-Up: Case Report and Review of the Literature

**DOI:** 10.3390/diagnostics12020342

**Published:** 2022-01-28

**Authors:** Milda Sarkinaite, Indre Devyziene, Jurgita Makstiene, Algimantas Matukevicius, Rymante Gleizniene

**Affiliations:** 1 Department of Radiology, Lithuanian University of Health Sciences, 44307 Kaunas, Lithuania; rymante.gleizniene@lsmuni.lt; 2 Department of Radiology, Hospital of Lithuanian University of Health Sciences Kauno Klinikos, 50161 Kaunas, Lithuania; indre.matukeviciute@kaunoklikos.lt; 3 Department of Pathology, Lithuanian University of Health Sciences, 44307 Kaunas, Lithuania; jurgita.makstiene@lsmuni.lt; 4 Department of Neurosurgery, Lithuanian University of Health Sciences, 44307 Kaunas, Lithuania; algimantas.matukevicius@lsmuni.lt

**Keywords:** leptomeningeal spread, diffuse leptomeningeal glioneuronal tumour, central nervous system, paediatric

## Abstract

In 2016, the World Health Organisation Classification (WHO) of Tumours was updated with diffuse leptomeningeal glioneuronal tumour (DLGNT) as a provisional unit of mixed neuronal and glial tumours. Here, we report a DLGNT that has been re-diagnosed with the updated WHO classification, with clinical features, imaging, and histopathological findings and a 9-year follow-up. A 16-year-old girl presented with headache, vomiting, and vertigo. Magnetic resonance imaging (MRI) demonstrated a hyperintense mass with heterogenous enhancement in the right cerebellopontine angle and internal auditory canal. No leptomeningeal involvement was seen. The histological examination revealed neoplastic tissue of moderate cellularity formed mostly by oligodendrocyte-like cells. Follow-up MRI scans demonstrated cystic lesions in the subarachnoid spaces in the brain with vivid leptomeningeal enhancement. Later spread of the tumour was found in the spinal canal. On demand biopsy samples were re-examined, and pathological diagnosis was identified as DLGNT. In contrast to most reported DLGNTs, the tumour described in this manuscript did not present with diffuse leptomeningeal spread, but later presented with leptomeningeal involvement in the brain and spinal cord. Our case expands the spectrum of radiological features, provides a long-term clinical and radiological follow-up, and highlights the major role of molecular genetic testing in unusual cases.

## 1. Introduction

In 2016, the World Health Organisation (WHO) Classification of Tumours of the Central Nervous System (CNS) was updated with the inclusion of diffuse leptomeningeal glioneuronal tumours (DLGNTs) as a provisional unit of mixed neuronal and glial tumours, after an increasing number of cases reported on tumours observed seeming both neurocytoma-like and containing variable glial components, leading to a conclusion of a new type of neuronal–glial tumours [1,2,3,4]. Due to the limited number of reported cases, the frequently partial clinical follow-up and the variability of endpoint, DLGNTs have not yet been assigned a WHO grade. These tumours have been mainly identified in the paediatric population, with a median age of 5 years and a steadily accelerating course [5].

Molecular genetic studies often pose a combination of KIAA1549 and the serine/threonine protein kinase B-raf (BRAF) gene (KIAA1549-BRAF fusion), as well as the short arm of chromosome 1 (1p) and/or the long arm (19q) of chromosome 19 deletions [5,6]. The most prevalent imaging characteristic is the preponderant diffuse abnormal cystic or nodular leptomeningeal growth without an affirmation of a primary intraparenchymal focus [7,8]. The distinctive diffuse abnormal leptomeningeal growth with small, nodular T2W-hyperintense lesions resembling cysts is usually observed in the basal cisterns and posterior fossa and alongside the spinal cord. Prospective tumour identification via imaging is encumbered by a variety of histological, radiological, and clinical features and the very low range of occurrence.

Here, we report a case of DLGNT that has been re-diagnosed with the updated WHO classification, with clinical features, imaging, and histopathological findings, as well as a 9-year follow-up period, including literature research. Seeking to raise awareness of this novel entity, we also hope to ease rapid diagnosis in affected patients, and provide knowledge on the biological behaviour of this neoplasm over time.

## 2. Case Presentation

**Figure 1:**
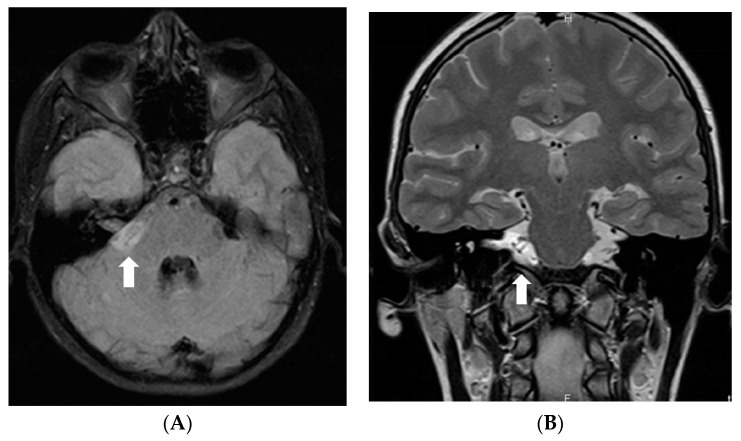

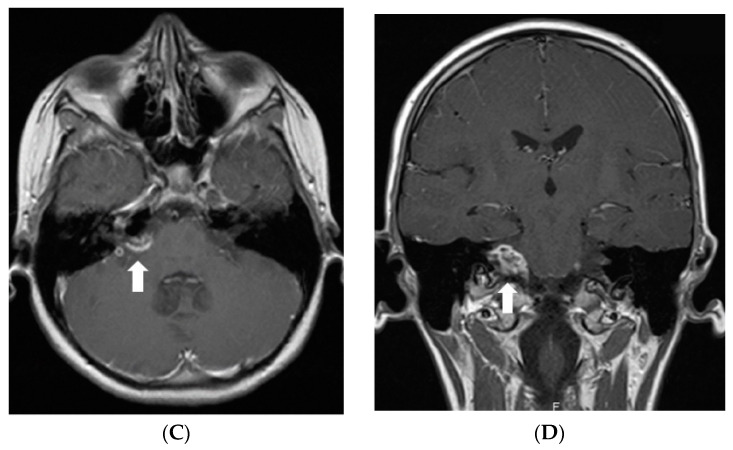
Initial MRI of the brain at our clinic (July 2011): (**A**) Axial T2W/FLAIR and (**B**) coronal T2W images demonstrate a hyperintense mass in the right cerebellopontine angle cistern and internal auditory canal. (**C**) Axial and (**D**) coronal T1W postcontrast images show heterogenous enhancement in the referred area.

In July 2011, a 16-year-old girl was admitted to Hospital of Lithuanian University of Health Sciences Kauno klinikos due to severe headache, vomiting, and vertigo lasting 2 days. She had a half-year history of similar paroxysms usually beginning with numbness in hands or in one side of face or leg. Neurological examination revealed right-sided peripheral facial paresis, horizontal nystagmus, painful exit points of branches of the right trigeminal nerve, slight deviation of the uvula to the right, right-sided hemiparesis, and cerebellar ataxia. Moreover, papilledema was observed. Brainstem evoked potentials response audiometry showed no clear waves on the right side although hearing was normal. Magnetic resonance imaging (MRI) of the head demonstrated a hyperintense mass on T2W/FLAIR images with heterogenous enhancement in the right cerebellopontine angle cistern and internal auditory canal (Figure 1). All figures in this manuscript are non-published and original.

During surgery, the tumour was found growing from the brainstem, inferoanterior margin of foramen Luschka, invading the proximal part of the right facial nerve, surrounding the right cranial nerve VIII, also extending to right cranial nerves V, IX, and X. The tumour was removed along with resection of the invaded part of the facial nerve. The histopathological examination revealed neoplastic tissue of moderate cellularity formed mostly by oligodendrocyte-like cells in sparse myxoid stroma. After surgery, total right-sided facial palsy developed as well as hearing having deteriorated in the right ear. A few months later, reconstructive surgery of the facial nerve was performed by creating hypoglossal-facial nerve anastomosis with sural nerve interposition graft. 

**Figure 2:**
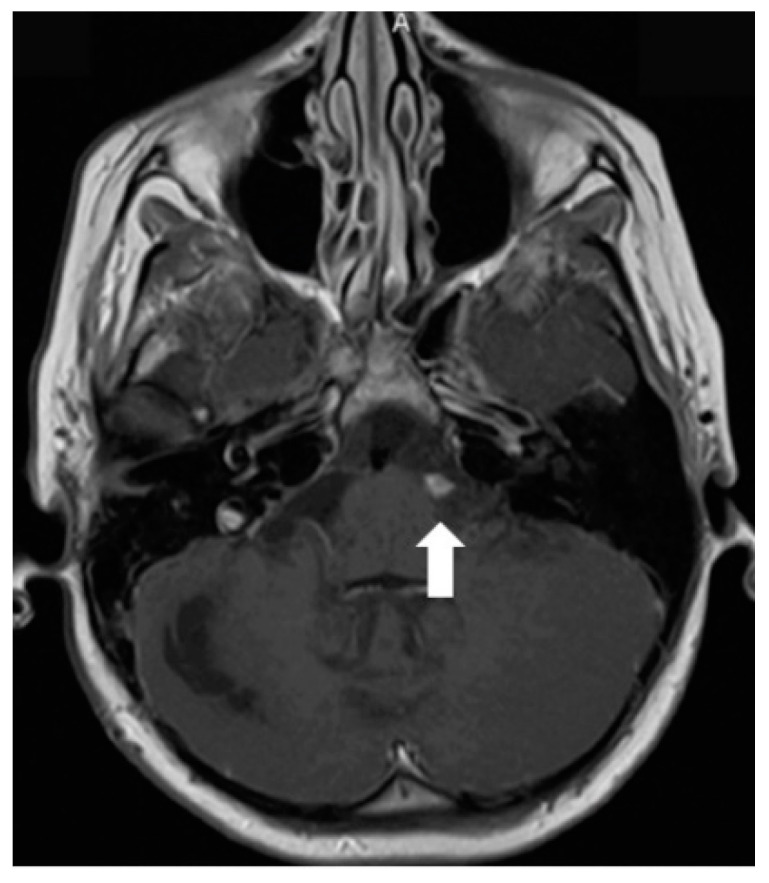
Follow-up MRI of the brain after the surgery (in December 2011). Axial post-contrast T1W image demonstrates a new enhancing nodule in the expected location of the cisternal segment of the left abducens nerve.

Five months after the first MRI, another MRI was performed, and it showed a new small enhancing nodule in the prepontine cistern at the pontomedullary junction, in the expected location of the left abducens nerve (Figure 2).

**Figure 3:**
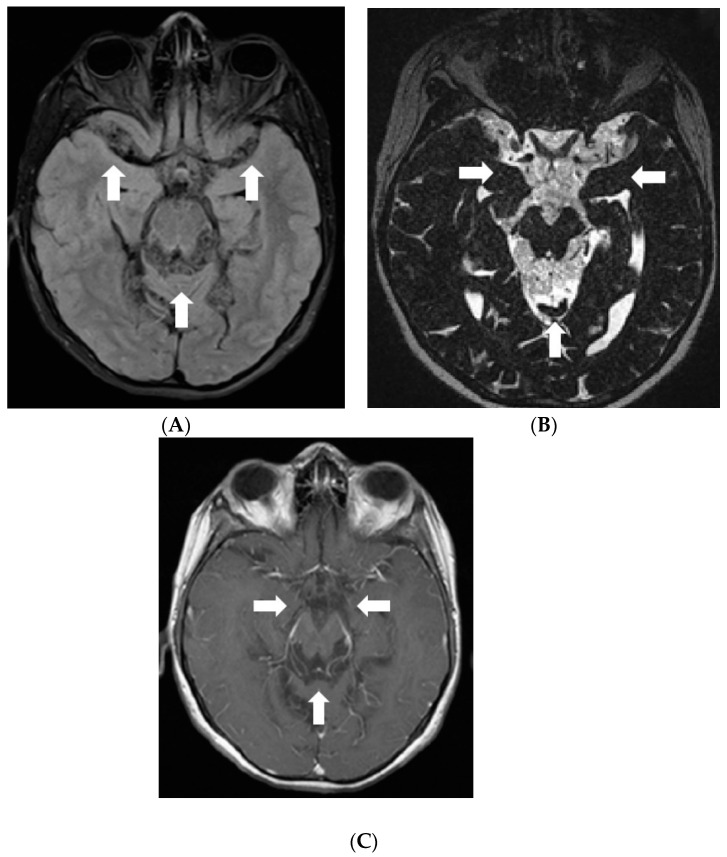
Follow-up MRI of the brain 1 year after the onset of the symptoms (April 2012): (**A**) Axial T2W/FLAIR/FS and (**B**) T2W/SPC images disclose masses composed of multiple small cysts in the subarachnoid cisterns, Sylvian fissures, and cerebellar sulci. There is no contrast enhancement (**C**) in the referred areas.

After 4 months when symptoms of raised intracranial pressure recurred, additional findings were detected on follow-up MRI. Most of the subarachnoid cisterns, Sylvian fissures, sulci of superior parts of the cerebellum, and the lower part of the fourth ventricle were filled with diffuse non-contrast-enhancing multiple small cyst-like lesions (Figure 3). These lesions were more evident on T2W/FLAIR and T2W/SPC images; T2W/FLAIR revealed iso/slightly hypointense lesions resembling cysts in the previously mentioned areas. The lesions did not show diffusion restriction or any significant compression of adjacent structures. Additionally, magnetic resonance spectroscopy of the lesion demonstrated lactate peak. Due to the uncertain origin of the findings, a biopsy of the newly detected tissue in the perimesencephalic cistern was performed. Histological appearance and immunohistochemical phenotype of the sample were the same as in the previous examination, consistent with subarachnoid spread.

The girl underwent induction chemotherapy according to low-grade glioma protocol (with vincristine, carboplatin, and etoposide); however, it was not effective and local radiotherapy was administered. Due to tumour-induced disorders of CSF reabsorption and further progressive hydrocephalus, a ventriculoperitoneal shunting was performed. Despite this measure, the patient’s status worsened as cerebellar ataxia, weakness of lower limbs, bulbar symptoms started to increase.

**Figure 4:**
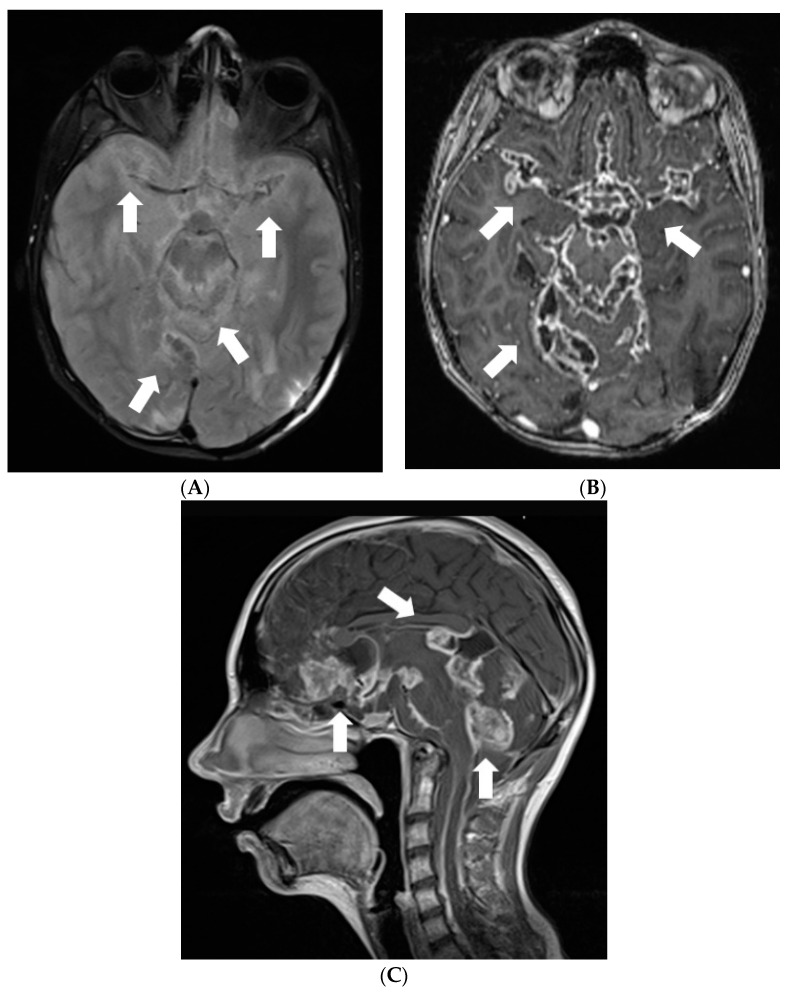
Follow-up MRI of the brain 2 years after the onset of symptoms (May 2013): (**A**) Axial T2W/FLAIR/FS, (**B**) axial, and (**C**) sagittal T1W postcontrast images demonstrate enlarged cystic lesions in the subarachnoid spaces causing compression of the brainstem and the fourth ventricle and vivid leptomeningeal enhancement extending to the spinal canal.

Later the girl was treated in Klaipeda University Hospital. In May 2013, a follow-up MRI demonstrated enlarged cystic lesions in the subarachnoid spaces with compression of the brainstem and the cerebellum and vivid leptomeningeal enhancement extending to the spinal canal (Figure 4). T2W/FLAIR/FS images revealed heterogenous, slightly hyperintense lesions with hypointense foci in the subarachnoid spaces. Clinically, ataxia and weakness of the legs worsened. Partial resection of the neoplastic masses from premedullary cisterns and the IVth ventricle was performed. It was decided to administer chemotherapy with temozolomide. Gradually, the condition of the patient improved.

**Figure 5:**
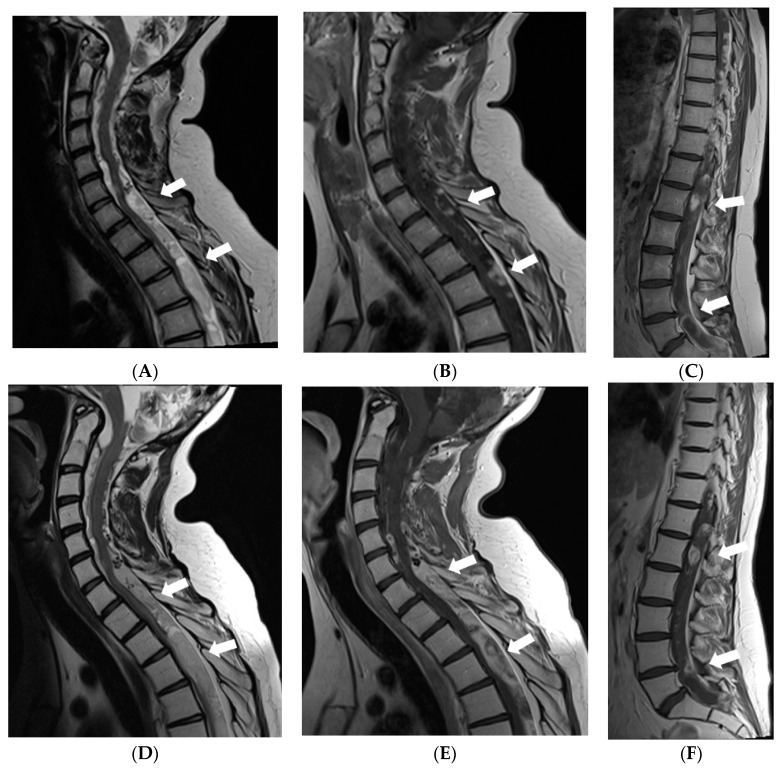
MRI of the spinal cord 6 years after the onset of symptoms: (**A**,**D**) Sagittal T2W, (**B**,**E**) T1W postcontrast images of the cervical and the upper thoracic spine, (**C**,**F**) postcontrast images of the middle/lower thoracic and the lumbar spine. In May 2017, (**A**–**C**) multiple confluent cystic extramedullary masses were found in the spinal canal through all its length, compressing the spinal cord at the level from C6 to T9 vertebrae. There was some nodular contrast-enhancement in the tumourous masses and diffuse meningeal enhancement. After radiotherapy in December 2017 follow-up MRI (**D**–**F**) demonstrated enlargement of the extramedullary masses in the lower cervical-upper thoracic spine with more vivid and diffuse enhancement, also, more vivid diffuse meningeal enhancement.

In 2017, follow-up brain MRI scans showed decreased leptomeningeal enhancement and slight decrease in size of cystic lesions in the brain; although, atrophy of the brainstem and cerebellum had progressed. However, spread of the tumour was found in the spinal canal through all its length manifested by diffuse meningeal enhancement and multiple cystic intra- and extradural lesions causing compression of the spinal cord. The girl received radiotherapy to the whole spinal cord; however, masses in the spinal canal progressed (Figure 5). In 2018, right-sided facial reconstructive surgery with medial cantopexy was performed.

**Figure 6:**
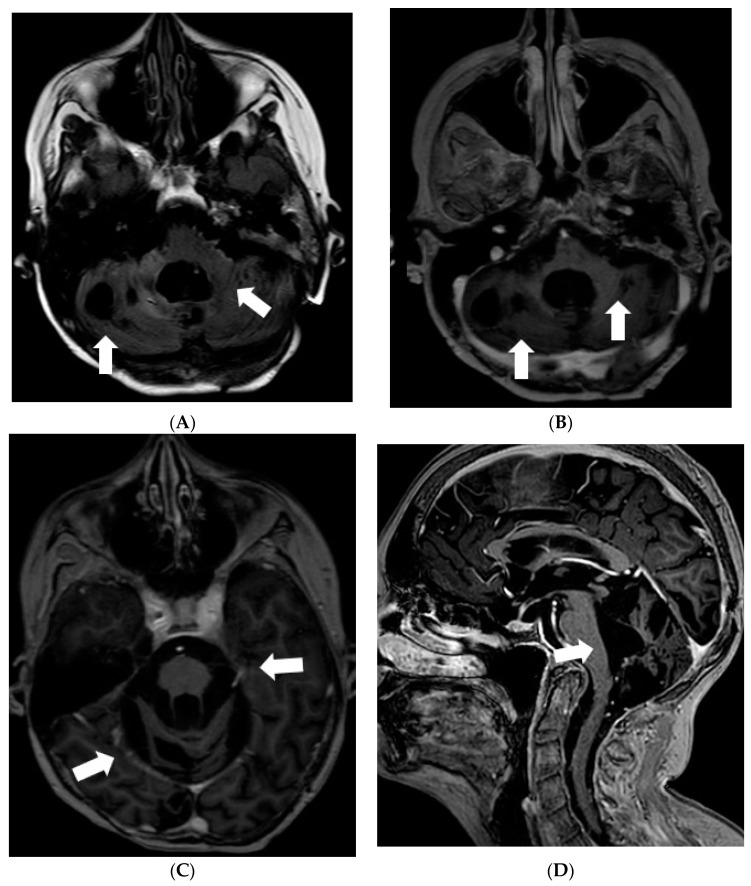

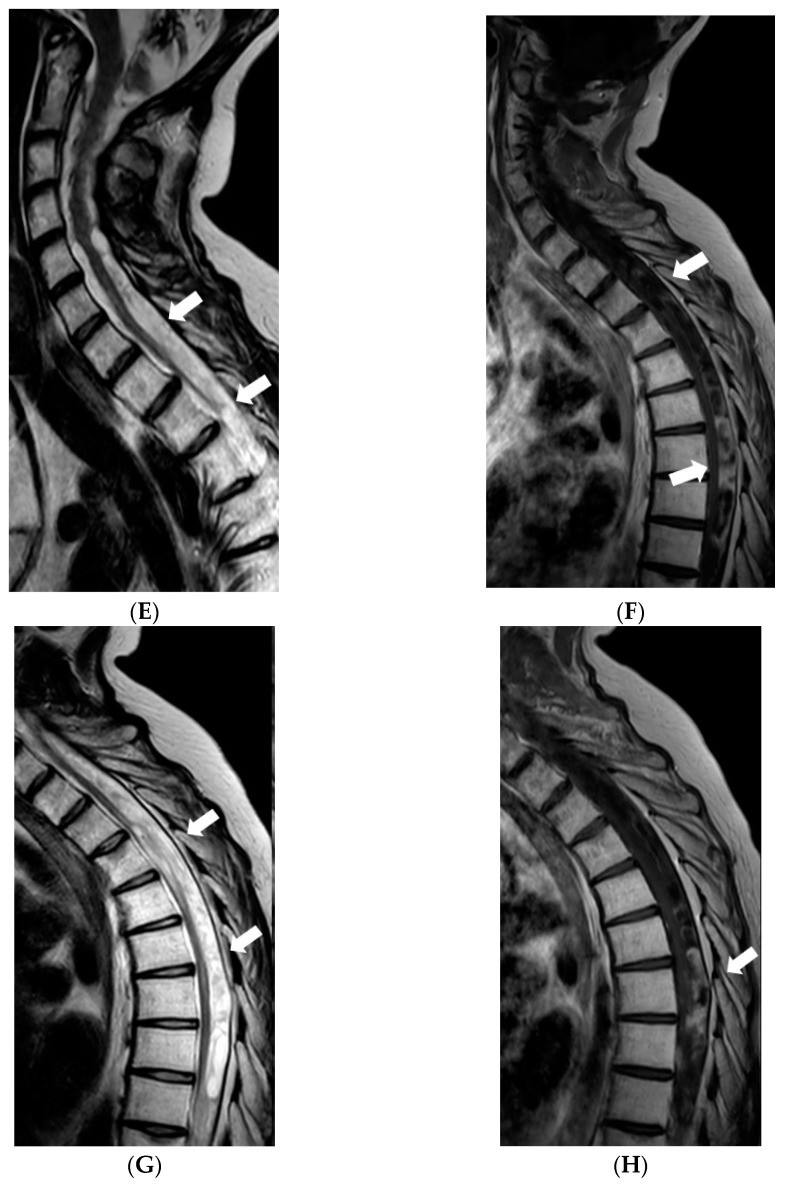

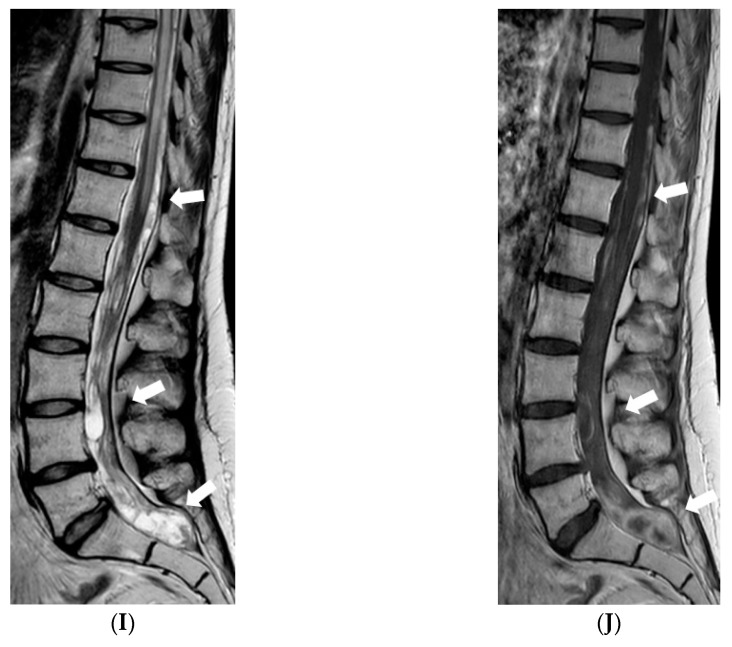
Follow-up MRI of the brain and spinal cord 9 years after the onset of symptoms (November 2020): (**A**) Axial T2W/FLAIR, (**B**,**C**) and (**D**) sagittal T1W post-contrast images demonstrate residual non-enhancing cystic lesions in basal cisterns and posterior fossa surrounding brainstem and cerebellum. (**D**) Sagittal T1W post-contrast image shows diffuse atrophy of the cerebellum. (**E**,**G**,**I**) Sagittal T2W and (**F**,**H**,**J**) T1W post-contrast images of (**E**,**F**) cervical, (**G**,**H**) thoracic, and (**I**,**J**) lumbar spinal cord reveal diffuse intradural extramedullary mixed cystic and solid lesions that show medium contrast enhancement and slight compression of spinal cord at various levels.

Due to impaired vision, further loss of hearing, and ability to walk, an MRI of the brain and spine was repeated in 2020. MR brain revealed residual changes—cystic leptomeningeal-subpial nodular lesions that were hypointense on T2W/FLAIR, and did not show contrast enhancement in the posterior fossa and along the basal cisterns, surrounding the brainstem, cranial nerves, and cerebellum. Imaging of spinal cord revealed cystic nodular lesions resembling those found in the brain and causing persistent, multisegmental spinal cord compression (Figure 6). 

**Figure 7:**
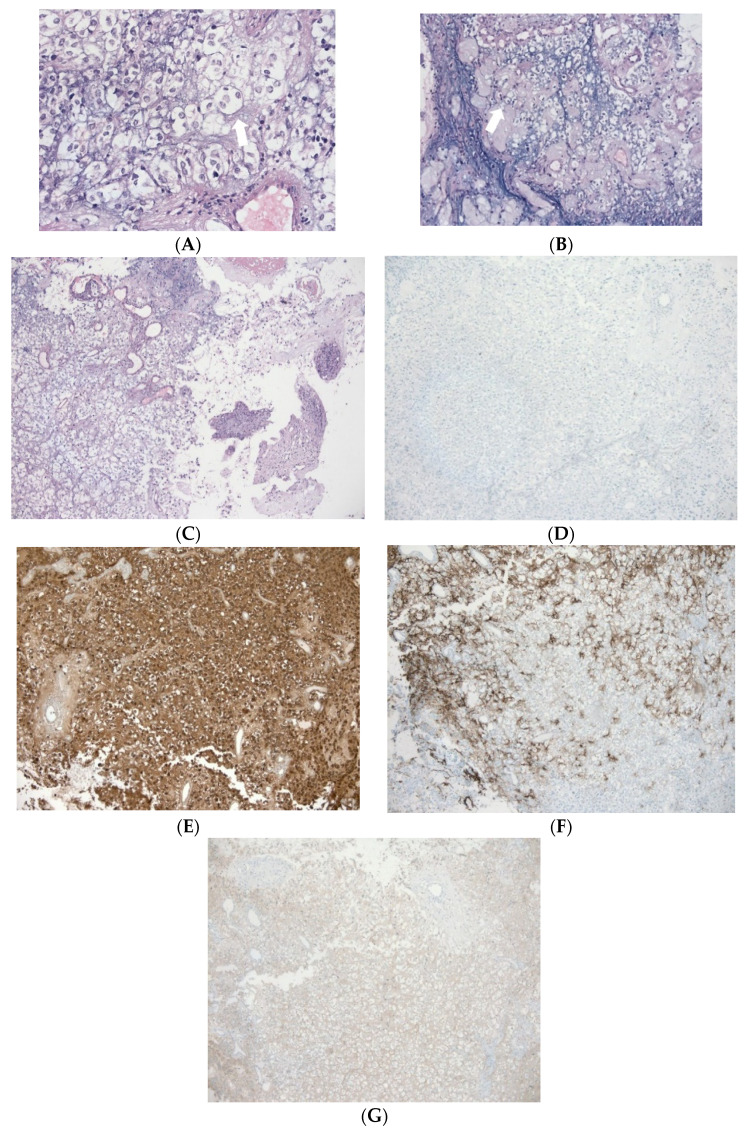
Histological features and immunohistochemical profile of the tumour: (**A**–**C**) H&E-stained sections demonstrate monotonous oligodendrocyte-like or neurocyte-like tumour cells with round nuclei and clear cytoplasm. Note none microvascular proliferation or significant mitoses in the neoplasm. (**D**) KI67 labelling index was around 2%. (**E**) The tumour cells are strongly immunopositive for S-100 and (**F**) weakly reactive for GFAP and (**G**) synaptophysin. Original magnification: ×100 for (**C**–**E**,**G**); ×200 for (**B**,**F**); ×400 for (**A**).

On demand biopsy samples removed from the tissues of the brain surface in 2012 and 2013 and the Sylvian fissure leptomeninges were re-examined. The pathological examination disclosed a tumour composed of comparatively monomorphic oligodendrocyte-like cells with moderately sized round nuclei and nucleoli and perinuclear halos. The tumour cells formed large accumulations in the leptomeninges with hyalinized small blood vessels around them. There were neither neurocytic rosettes or foci of necrosis, nor microvascular proliferation. No significant mitoses were observed. The Ki67 proliferation index proved to be low (2%). Immunohistochemistry showed that the tumour cells were highly reactive to S100, less reactive to glial fibrillary acidic protein (GFAP) and synaptophysin, and negative for epithelial membrane antigen (EMA) reactivity. IDH1 and IDH2 mutations proved to be negative. Fluorescence in situ hybridisation (FISH) examination showed a deletion of 1p36. The BRAF V600E expression (VE1 antibody) was not detected. Based on these findings, the pathological diagnosis was identified as DLGNT (of an unspecified grade) (Figure 7). 

## 3. Discussion

In the updated 2016 edition of WHO Classification of Tumours of the CNS, DLGNTs have been classified as a separate entity [9]. DLGNTs were labelled as neuronal and mixed neuronal–glial tumours due to their inherent “oligodendroglia-like” cells with a variable neuronal component (from neurocytes to ganglion cells) [10]. In 2021, the WHO Classification of Tumours of the CNS added diffuse glioneuronal tumours with oligodendroglioma-like features and nuclear clusters as a separate entity, but further studies are required for full acceptance [11]. This tumour is very rare. Since 2012, when the largest series of 36 patients was first reported by Rodrigues et al. [5], fewer than 100 cases of DLGNTs have been reported worldwide.

Although we had a DLGNT occurrence in a 17-year-old female patient in our case, research of the literature on published cases of DLGNTs shows that the median age of onset of a DLGNT approximates 4 years, with a male prevalence in 58 out of 91 cases (64%) [12,13,14]. Adult cases are sparse [15]. Clinical symptoms are not specific and commonly include headaches, vomiting, seizures, and cranial nerve palsy, as in our case [16,17]. As in many other cases of this disease, the clinical progression is slow, but some cases of anaplastic transformation have been reported [18,19].

Histologically, this lesion has the classic oligodendroglial pattern: round cells with surrounding halos [5]. Astrocytic features are also observed in a small number of cases [5]. On immunohistochemistry, tumour cells are positive for OLIG2, MAP2, S100, and less than 50% are immune-positive for GFAP. Synaptophysin, chromogranin A, and other neuronal markers may be positive in the cases with prominent neuronal signs [5]. In our case, immunostaining against GFAP and S-100 antibodies was positive, highlighting the glial origin, and positive synaptophysin staining was indicative of neuronal differentiation, confirming the findings of previous studies [5,20,21]. The Ki-67 (MIB1) labelling index reported ranging from <1% to 15%. In our case, the Ki-67 index was 2%, which is a good prognostic factor, as according to a review of the literature, a less favourable endpoint is associated with an index >4% [5,8,22]. Aggressive features such as necrosis, multiple mitotic figures, and microvascular proliferation are not typical [3]. On immunohistochemistry, the tumour cells reveal positive IDH1 (R132H), whereas EMA is negative, as in our case. Molecular alterations characteristic of this tumour includes concomitant KIAA1549-BRAF gene fusions, most of which are negative due to the BRAF V600E mutation, and sporadic deletion of 1p or codon deletion of 1p/19q in the absence of the IDH mutation [5]. Rodriguez et al. [6] also found that KIAA1549-BRAF fusion was positive in 75% of cases, suggesting an association with pilocytic astrocytoma. In our case, the FISH analysis was positive only for the 1p deletion and the BRAF V600E mutation was negative. In a previous study, loss of 1p/19q intact was the most frequent finding (50–60%), followed by absence of the 1p19q co-deletion (27%) and absence of the 1p19q co-deletion (20%) [5]. Studies have shown that 1p/19q co-deletion or deletion of either 1p or 19q alone is indicative of the biological behaviour of malignancies, suggesting that the patients were sensitive to chemotherapy, particularly temozolomide (TMZ) [12]. Recently, DNA methylation profiling has identified two molecular subgroups: MC-1, which is associated with a better prognosis; and MC-2, which is associated with a worse prognosis [23]. Despite the undetermined pathogenesis, pathological and genetic evidence supports the association of DLGNTs with pilocytic astrocytoma or glioneuronal tumours [1,8,14].

As in our case, leptomeningeal cystic changes may be absent in the early phase of the disease, thus further hampering the diagnosis. As the disease progressed, the DLGNT in our case presented with a classical imaging appearance. The most common MRI finding of DLGNTs that could serve as an important diagnostic marker of this disease is leptomeningeal growth involving the spinal cord and basilar cisterns, frequently accompanied by cystic T2W-hyperintense lesions alongside the basilar cisterns and subarachnoid space, often in the absence of a solid intraparenchymal brain tumour [13,24]. What is more, DLGNT in our case presented not only with cystic but also with solid components. Having examined 15 cases of DLGNTs, 7 patients had spinal involvement. These patients developed nodular intramedullary lesions before the development of cystic changes in the brain, raising the possibility that this lesion is a primary spinal cord tumour [12]. In another multicentre case series defining the molecular subgroups of DLGNTs, a detailed analysis of radiological data from 15 patients showed spinal involvement in 14 patients (93%) with superficial/subpial cysts and parenchymal involvement, and occasionally cerebellar involvement in 5/15 patients (33%) [25]. These reports suggest that spinal involvement is very common in DLGNTs. However, in a recent study by Chiang et al. involving five patients, leptomeningeal extension was not radiologically observed in any case, suggesting that DLGNTs do not necessarily present with gross leptomeningeal disseminations on MRI imaging [26]. In many cases [19,27,28,29], including ours, hydrocephalus may also have facilitated further tumour spread [30]. Hydrocephalus may have developed due to the tumour’s impaired reabsorption capacity and high CSF protein content [30]. In our case, despite the increased possibility of peritoneal spread, VP bypass was unpreventable.

One of the main features of DLGNTs is diffuse leptomeningeal growth, for which the differential diagnosis is extensive. The primary radiological diagnosis in our case was difficult because diffuse multicystic lesions in the brain did not show contrast enhancement. On the other hand, the most common cause of diffuse leptomeningeal enhancement is meningitis, with a variety of aetiologies. Of all the causes, bacterial and viral meningitis with thin linear meningeal enhancement is the most prevalent [30]. Following our literature research, DLGNTs are often misdiagnosed as cases of tuberculosis, especially in the regions of Europe and the Asia-Pacific region [2]. Medulloblastoma and other CNS embryonal tumours, ependymoma, germinoma, pineoblastoma, and high-grade radial glioma frequently cause carcinomatous meningitis [30]. Involvement of the meninges by secondary tumours is most commonly associated with malignancies originating from the lung and breast, lymphoproliferative disorders, and melanoma [31]. In moyamoya disease, diffuse leptomeningeal enhancement is scarce, which is caused by an engorged pial network via leptomeningeal anastomosis [32]. Other neoplasms characterised by diffuse leptomeningeal enhancement consist of primary diffuse leptomeningeal gliomatosis and diffuse leptomeningeal glioneuronal neoplasm [8,33]. Notwithstanding, the main imaging differential diagnosis of DLGNTs includes chronic infectious meningitis, tuberculous meningitis, sarcoidosis, leptomeningeal intraparenchymal diffuse astrocytoma, and pilocytic astrocytoma. Correlation with clinical characteristics of infection and assessment of the nature of the leptomeningeal lesion may help to distinguish DLGNTs from infection. Bacterial meningitis usually presents with smooth rather than nodular sulcal-cisternal growth, whereas tuberculous meningitis more commonly leads to confluent basilar growth. The vesicular stage of neurocysticercosis may manifest as multiple cysts appearing with internal distinctive fragmented ‘dots’ in deep sulci and characterised by an intense inflammatory reaction in the adjacent parenchyma, which is unusual for DLGNTs. Apart from noninfectious CSF findings, the most important feature to differentiate DLGNTs from other lesions is the presence of diffuse leptomeningeal lesions in the brain and spinal cord with or without multifocal cystic-like lesions or a solitary solid spinal mass.

DLGNTs are usually characterised by periods of stable disease and a slow course, with most patients surviving for decades, except for the cases of secondary hydrocephalus, which has a high morbidity rate [19,34,35]. However, prognosis of DLFNT between children and adult population remains unclear due to the rarity of this entity. What is more, there are not enough established treatment protocols for this disease, so comparison of clinical outcomes for patients on different treatment methods is not possible [36].

Despite the breakthrough in our knowledge of the diagnostic substantiation and clinical appearance of DLGNTs, there is still no consensus on the suitable perspective to treatment for these patients and reports on the treatment modalities being used vary widely. Chemotherapy with carboplatin and vincristine or temozolomide has been suggested as the first-line approach for this condition, with radiotherapy as a resort to the cases when the disease progresses [5,36]. Despite the typical indolent course, similar to patients with low-grade gliomas, these cases usually require use of multiple chemotherapy regimens over the lifetime of these patients. This implies the need for the development of new and possibly targeted therapeutic regimens for patients with DLGNTs.

## 4. Conclusions

In the light of the recent WHO classification amendment and the rarity of this entity, many radiologists and referring medical practitioners may not yet be aware of DLGNT. It commonly presents with diffuse abnormal leptomeningeal growth, but radiological findings may vary. Thus, substantiation of the diagnosis by molecular genetic means is an ideal approach. Our patient’s case expands the spectrum of radiological findings, and gives an opportunity for a long-term clinical and radiological follow-up, which contributes to our understanding of the conduct of DLGNTs, and highlights the major role of molecular genetic testing in unusual cases.

## Data Availability

The data presented in this study are available on request from the corresponding author. The data are not publicly available due to ethical restrictions.
